# Cross-sectional study of mechanism of arterial pulsation amplitude modulation under ultrasound probe compression: Similarities and differences compared with the oscillometric blood pressure measurement principle

**DOI:** 10.1097/MD.0000000000049218

**Published:** 2026-06-12

**Authors:** Takuya Ito, Ryohei Matsui, Marechika Tsubouchi, Mami Tsubota, Yota Yamagishi, Tomonori Hattori, Hiroshi Sasano

**Affiliations:** a Department of Advancing Acute Medicine, Nagoya City University Graduate School of Medical Sciences, Nagoya, Aichi, Japan.

**Keywords:** arteries, blood pressure determination, oscillometry, ultrasonography

## Abstract

Arterial pulsation is used to differentiate arteries from veins on ultrasound imaging. However, the mechanism by which the arterial pulsation amplitude changes when an ultrasound probe compresses the artery has not been reported. Here, we aimed to investigate whether this mechanism resembles the oscillometric maximum amplitude algorithm. An automatic blood pressure cuff was placed on the upper left arm of 7 healthy participants (four men and 3 women; aged 27–46 years). An ultrasound probe attached to a force sensor was gradually applied to the distal brachial artery using an automated test stand until the lumen of the artery was completely occluded. Probe pressure and arterial pulsation data were recorded and analyzed during compression. Sequential ultrasound images and probe pressure measurements were obtained over time under 2 conditions: without cuff occlusion and with the cuff inflated above the systolic blood pressure. Without cuff inflation, ultrasonography-detected arterial pulsations and probe pressure oscillations were perfectly synchronized. The timing of the maximum amplitude occurrences in both signals coincided. Conversely, when pulsatile flow was stopped by cuff inflation above the systolic pressure, neither arterial pulsation nor probe pressure oscillations were observed. Pulsatile oscillations in the probe pressure persisted after local arterial occlusion by the probe. The mechanism underlying arterial pulsation generation under probe compression can be explained by the transmural pressure–volume relationship of the artery, in which pulsatile flow and reduced transmural pressure cause changes in arterial volume and produce observed probe pressure oscillations. These findings link ultrasound imaging and oscillometric blood pressure principles, providing a unique perspective for noninvasive arterial hemodynamic assessment.

## 1. Introduction

**Figure 1.**
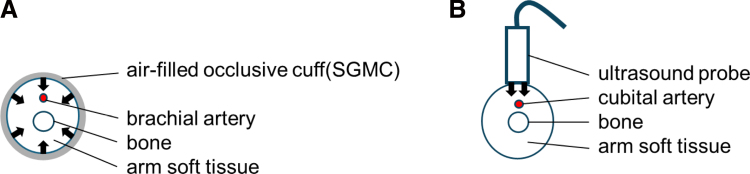
Differences in the effects of arterial compression between (A) sphygmomanometer cuff (SGMC) inflation and (B) probe-induced compression. The black arrows represent the direction of pressure applied by either the cuff or the probe. The pressure exerted by the SGMC is circumferential, whereas that applied by the ultrasound probe is unidirectional.

Arterial pulsation, along with arterial resilience relative to veins and characteristic color Doppler signals, is a key clinical indicator used to distinguish arteries from veins on ultrasound imaging.^[1]^ Oscillometric blood pressure measurements rely on the principle that pulsatile blood flow in the artery beneath the cuff, combined with changes in transmural pressure caused by cuff inflation, leads to variations in the arterial volume.^[2,3]^ These volume fluctuations, in turn, generate oscillations in the cuff pressure according to the arterial pressure–volume relationship.^[4,5]^ The gradual change in sphygmomanometer cuff pressure alters the arterial transmural pressure. When the cuff is inflated above the systolic blood pressure and then gradually deflated, the arterial transmural pressure shifts from negative to positive. At the point where the transmural pressure reaches zero, the compliance of the arterial wall becomes maximal.^[3]^ The resulting pulsatile blood flow induces corresponding fluctuations in the arterial volume, which manifest as oscillations in the cuff pressure. By analyzing these oscillations, the point of maximum amplitude, representing peak arterial compliance, is identified as the mean arterial pressure.^[4–8]^ This is the fundamental principle of the maximum amplitude algorithm.^[6,7,9]^ However, the oscillometric principle relies on cuff-based compression, and it remains unknown whether this principle holds true under different compression conditions, such as compression by an ultrasound probe (Fig. 1). In our previous research, we demonstrated that compressing the arterial systolic diameter from 75% to 50% using an ultrasound probe increased the amplitude of arterial pulsation.^[1]^ However, the underlying mechanism responsible for this effect has not been elucidated. Furthermore, no previous studies have simultaneously recorded the force measured at the probe and the resulting changes in pulsation amplitude on ultrasound imaging in a quantitative manner. Therefore, in this study, we aimed to determine whether changes in arterial pulsation amplitude under ultrasound probe compression follow a mechanism analogous to that of oscillometric blood pressure measurement.

**Figure 2.**
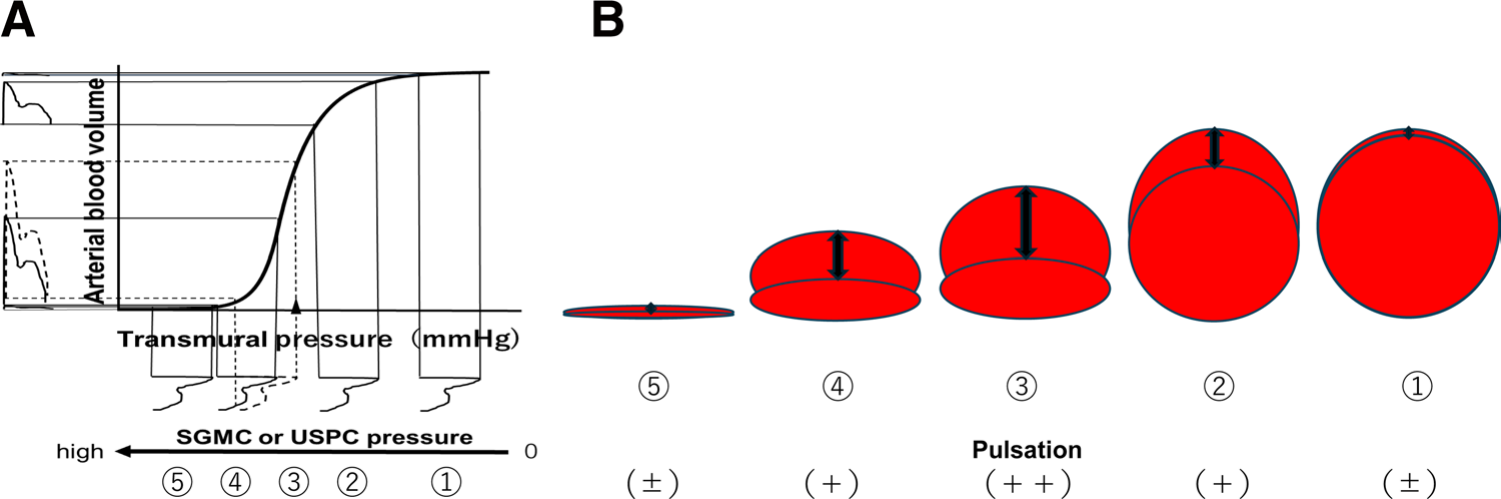
Arterial transmural pressure–volume relationship curve and probe-induced arterial compression. The left panel (A) illustrates the relationship between arterial volume and transmural pressure for both sphygmomanometer cuff (SGMC) pressure and ultrasound probe compression (USPC). When the transmural pressure (mean arterial pressure minus cuff or probe pressure) reaches zero (black triangle in the left panel [A]), the arterial compliance is maximized, as indicated by point ③ on the right side of the diagram (panel [B]). The right panel (B) illustrates the corresponding changes in arterial shape and pulsation amplitude during progressive compression.

We hypothesized that the mechanism of fluctuation in arterial pulsation under ultrasound probe compression resembles that of the oscillometric principle. Specifically, we propose that probe-induced changes in arterial transmural pressure, combined with pulsatile blood flow, produce simultaneous changes in arterial volume (diameter) and pulsatile fluctuations in probe pressure, consistent with the arterial pressure–volume relationship (Fig. 2).

## 2. Methods

This cross-sectional observational study was based on previous experimental data.^[1]^ The study protocol complied with the Declaration of Helsinki and was approved by the Nagoya City University Graduate School of Medical Sciences (Nagoya, Aichi, Japan) Review Board (No. 60-25-0027). Seven healthy adult volunteers (four men and 3 women, aged 27–46 years; height, 154–174 cm; weight, 48–75 kg) participated in the study. The inclusion criteria were age <60 years and no history of hypertension, arrhythmia, diabetes, or other relevant medical conditions. All participants provided written informed consent after receiving a verbal explanation of the study procedures.

**Figure 3.**
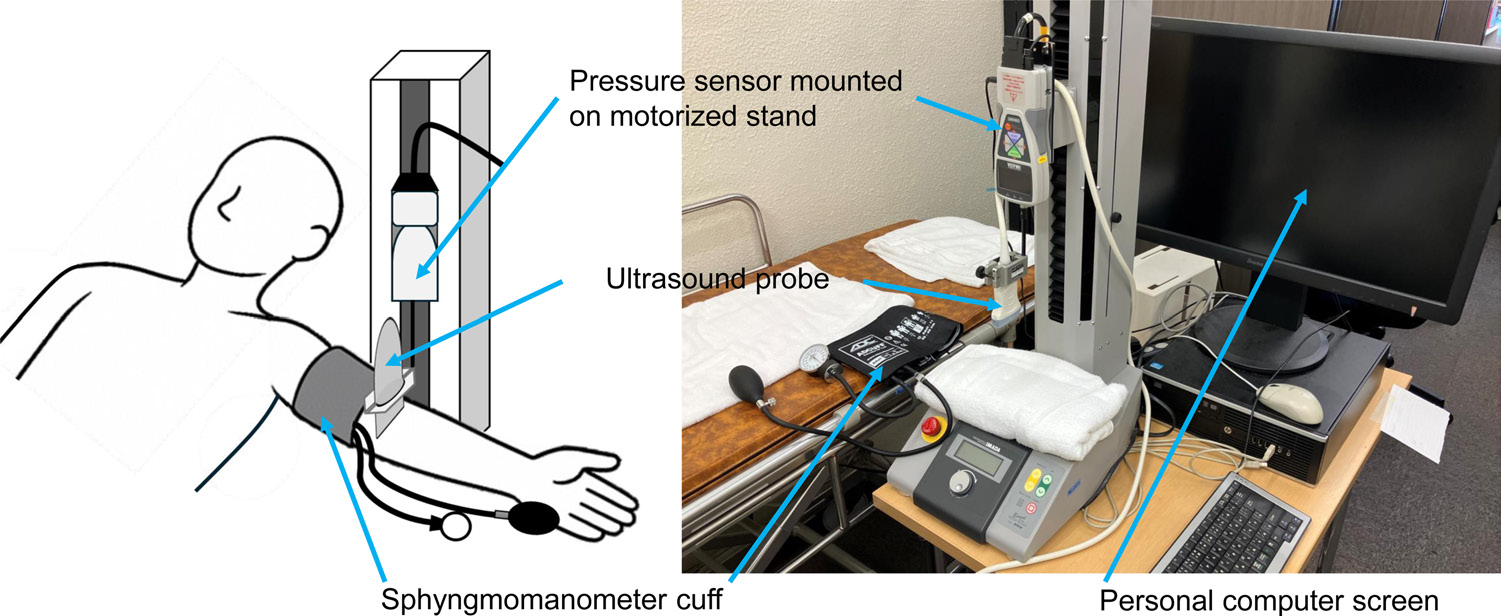
Experimental setup. Blue arrows indicate the devices used in this study.

The study was performed under stable indoor conditions, with ambient temperature maintained at 24°C to 25°C, and relative humidity between 50% and 60%, between April and October 2022. Using the existing dataset (Fig. 3), we selected the ultrasound and pressure recordings under 2 conditions: no cuff inflation (sphygmomanometer cuff pressure, 0 mm Hg) and cuff inflation at systolic blood pressure plus 20 mm Hg.^[1]^ Transverse ultrasound cross-sectional images of the brachial artery and adjacent veins at the antecubital fossa were obtained using the LOGIQ E9 ultrasound system (GE Healthcare, USA). To measure the probe pressure, we attached a linear 11.0 MHz ultrasound probe (GE Healthcare, USA) to a digital force gauge (Imada ZTA-50N, Japan) mounted on a holder (MP-PH0001-2, ALOKA, Japan). The assembly was mounted on an electric test stand (Imada EMX-1000N, Japan) and programmed to descend at a constant speed of 12.5 mm/min, applying pressure to the brachial artery in the left antecubital fossa through the skin. The artery was compressed within the central 3 cm of the 5 cm wide linear ultrasound probe. The total compression time to arterial occlusion ranged from 60 to 120 seconds. For analysis, the time point at which the probe was fully released from the skin surface was defined as time zero. Probe pressure data were recorded at 1000 Hz on a computer using an Imada Force Recorder Professional version 1.03 (Imada, Japan). Simultaneously, an ultrasound examination was performed at 30 Hz using an HD PVR video recorder (Rocket model 1536, Hauppauge, USA). Two investigators (TI and HS) independently analyzed the recorded ultrasound videos using ImageJ version 1.53t (Image Processing and Analysis in Java) by frame-by-frame tracking of the arterial diameter. Any discrepancies between the 2 measurements were resolved through discussion and consensus. To visualize the temporal changes in arterial pulsation amplitude and probe pressure amplitude, we generated M-mode–like images from ultrasound recordings using the Reslice function in ImageJ.

### 2.1. Measurement of pulsation amplitude

On ultrasound images, we identified each arterial pulse by its diameter peak and trough and recorded these values to generate a time series of arterial pulsation. The amplitude of each pulsation was defined by drawing a baseline between the troughs immediately before and after the peak and measuring the perpendicular height from this line to the peak. Similarly, we visually identified the peaks and troughs in the probe pressure recording to construct a time series of probe pressure oscillations. The amplitude of each probe pressure oscillation was measured as the difference between the peak and baseline drawn through neighboring troughs following the same procedure.

### 2.2. Analysis of synchrony

For each condition, we examined whether the oscillatory peaks of the arterial pulsation and probe pressure were synchronized. Arterial diameter was measured until the lumen was no longer visible on ultrasound imaging. Then, the timing of the peaks in both the arterial pulsation amplitude and the pulsatile component of the probe pressure was analyzed to assess their temporal alignment.

### 2.3. Outcome measures

The primary outcome was to determine: whether fluctuations in probe pressure are synchronized with arterial pulsations observed on ultrasound under nonocclusive conditions; whether the timing of maximal pulsation amplitude is identical between the 2 signals; and whether arterial pulsations disappear when pulsatile flow is occluded using a sphygmomanometer cuff. The secondary outcome was to evaluate whether pulsatile fluctuations in probe pressure and ultrasound imaging persist under complete arterial occlusion with sphygmomanometer cuff pressure.

### 2.4. Statistical analysis

Rather than conducting hypothesis testing, we summarized the difference in peak timing using the mean, standard deviation, median, and 95% confidence interval. All descriptive analyses were performed using R version 4.5.1 (R Foundation for Statistical Computing, Vienna, Austria). This descriptive approach was selected to assess whether the observed differences were clinically negligible and remained within a predefined acceptable range (±1.0 beats).

## 3. Results

**Figure 4.**
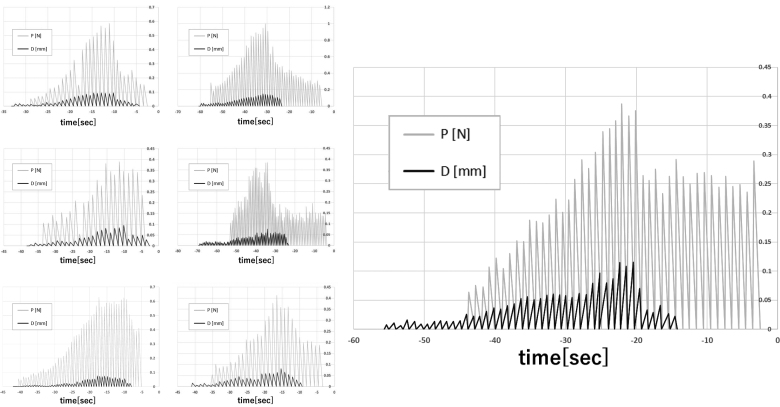
Time course of probe pressure (P) and arterial diameter (D) on ultrasound imaging under a sphygmomanometer cuff pressure of 0 mm Hg. The time point at which the probe was fully released from the skin surface was defined as time zero. The mean timing difference between the ultrasound-detected arterial pulsation peaks and the corresponding probe pressure oscillation peaks was .14 beats (standard deviation: .90 beats), with a 95% confidence interval ranging from −69 to + .98 beats.

**Figure 5.**
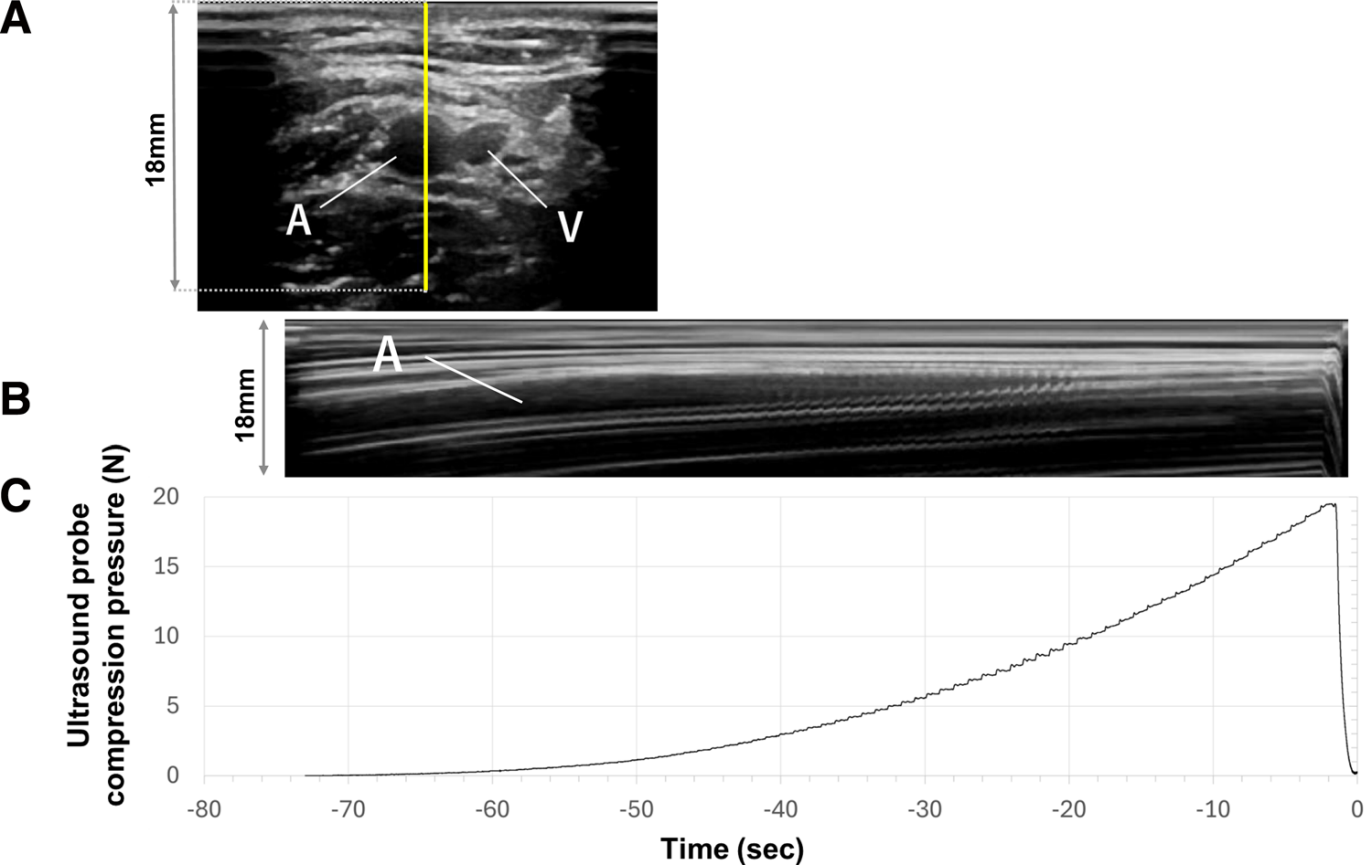
Time course of arterial pulsation under a sphygmomanometer cuff pressure of 0 mm Hg. **Top panel (A**) The ultrasound image shows the artery (A) and vein (V). The yellow line indicates the cut line used to generate the M-mode-like image in panel (B) via the Reslice function in ImageJ (version 1.53t); it was placed at a depth of 18 mm from the probe surface. **Middle panel (B):** A time series image constructed using the Reslice function in ImageJ, producing an M-mode-like representation of arterial wall motion along the cut line shown in panel (A). **Bottom panel (C):** Time course of the pressure applied by the probe.

Under nonocclusive conditions (sphygmomanometer cuff pressure of 0 mm Hg), superimposition of the probe pressure amplitude waveform and the arterial pulsation amplitude waveform revealed a characteristic modulation pattern, with probe pressure fluctuations remaining closely synchronized with the arterial pulsations. In all participants, as probe pressure increased, the pulsation amplitude initially rose, reached a relatively distinct maximum at an intermediate level, and subsequently decayed. Additionally, the peaks of the arterial pulsation visualized by ultrasound and the oscillations detected in the probe pressure appeared in close temporal proximity in every participant (Fig. 4). Quantitative analysis showed a minimal phase difference between the arterial pulsation and probe pressure peaks (mean,–.14 beats; standard deviation,.90 beats; median 0, 95% confidence interval,–.69 to .98 beats), supporting the visual impression of synchrony between the waveforms. Furthermore, even after the artery directly beneath the probe was completely occluded, pulsatile fluctuations in probe pressure persisted (Fig. 5). This phenomenon likely results from pulse pressure conduction from the central and lateral arterial segments that were not directly compressed by the probe, which continued to transmit the pulsatile force to the probe sensor. These findings suggest that the pulsatile component of the probe pressure waveform corresponds closely to the arterial pulsation observed on ultrasound, likely reflecting the same underlying physiological event. However, the methods used to alter the transmural pressure differ between the sphygmomanometer and ultrasound probes. The cuff applies broad circumferential compression around the entire limb, whereas the ultrasound probe applies localized unidirectional pressure only at the point of contact. Nonetheless, both types of external pressures generate pulsatile pressure oscillations. Conversely, when the cuff was inflated to a systolic blood pressure plus 20 mm Hg, pulsatile flow ceased, and we observed neither arterial pulsation on ultrasound nor oscillations in the probe pressure in any participant (see Video, Supplemental Video, which shows a representative ultrasound recording with 2 adjacent vessels in the center of the screen, where the artery collapses first). These observations provide direct evidence that pulsatile flow within the artery is essential for generating both types of oscillations.

**Video 1.** 

## 4. Discussion

In this study, we assessed whether changes in arterial pulsation amplitude under probe compression follow a mechanism analogous to that of the oscillometric blood pressure measurement. The principle of maximum amplitude of cuff pressure oscillations representing peak arterial compliance has been supported by theoretical models and indirect pressure measurements; however, to our knowledge, no previous study has provided direct visual confirmation of this peak compliance point by simultaneously observing both the ultrasound-visible arterial pulsation amplitude and probe pressure oscillations.^[2,5,6,10]^ Our results demonstrate a clear temporal and amplitude correspondence between these 2 modalities. This supports the theoretical pressure–volume relationship proposed in prior studies and extends it by offering real-time, image-based verification.

Furthermore, this is the first study to show that external compression of an artery with an ultrasound probe can induce simultaneous fluctuations in both ultrasound-visible arterial pulsation and the pulsatile component of probe pressure via changes in transmural pressure. 2 key observations in the study supported our hypothesis. When pulsatile flow was present (i.e., without cuff occlusion), arterial pulsation was observed on ultrasound imaging. When pulsatile flow was eliminated by inflating the cuff to a systolic blood pressure plus 20 mm Hg, arterial pulsation disappeared on ultrasonography. The same principle applies to the probe pressure oscillations. These findings suggest that a pulsatile flow is essential for generating ultrasound-detectable pulsations and pressure oscillations. Additionally, during slow compression with the ultrasound probe, the oscillatory component of the probe pressure was synchronized with the arterial pulsation observed on the ultrasound. In all participants, the timing of the maximum amplitude pulses was nearly identical for both signals.

These results resemble the essential features of the oscillometric algorithm, in which gradual cuff inflation modulates the transmural pressure, resulting in pulsatile changes in the arterial volume that manifest as oscillations in the cuff pressure. The point of maximum oscillation corresponded to peak compliance and was used to determine the mean arterial pressure. Similarly, in our ultrasound-based observations, probe-induced compression altered the transmural pressure and generated corresponding oscillations in both the arterial diameter and probe pressure, suggesting the same underlying mechanism.

Notably, unlike oscillometric devices that infer pulsation indirectly from cuff oscillations, in the current study, we directly visualized actual arterial pulsations using ultrasound. While we considered whether non-flow-related mechanisms such as vasomotion (i.e., intrinsic arterial rhythmic activity independent of pulsatile flow) could explain the pulsations, our findings clearly demonstrated that in the absence of pulsatile flow, no arterial pulsations were observed on ultrasound. This provides visual evidence that the observed pulsations originated from flow-dependent mechanisms.

### 4.1. Similarities between sphygmomanometer cuff inflation and ultrasound probe compression

Both the oscillometric cuff inflation and ultrasound probe compression produced a peak oscillation amplitude in the measured pressure signal. In our ultrasound experiments, the pressure oscillation waveform recorded by the probe during slow arterial compression matched the waveform used in the oscillometric maximum amplitude algorithm (i.e., a rising–falling oscillation with a clear maximum). In each case, the peak oscillation corresponded to the timing of the maximal arterial pulsation amplitude.

### 4.2. Differences between sphygmomanometer cuff inflation and ultrasound probe compression

The arterial diameter measurement reflects only the vessel directly beneath the probe, whereas the probe pressure reading is influenced by the pulsations of nearby and target arteries. This explains why pulsatile pressure oscillations remained after local occlusion by the probe (conducted from the proximal and lateral arterial trees). In addition, ultrasound probe compression primarily affects the artery under the probe via unidirectional (normal) pressure, whereas a cuff applies circumferential compression to the entire limb. These differences may alter how pressure is transmitted, thereby warranting further investigation.

Notably, we measured probe compression corresponding to cuff inflation, whereas conventional oscillometric methods typically measure compression during cuff deflation. Inflation-based oscillometric measurement has also been proposed to reduce measurement time without compromising accuracy.^[11,12]^

### 4.3. Clinical implications

Ensuring pulsatile flow is essential from a practical standpoint. For example, the partial occlusion of an artery by a probe (compressing to approximately half the lumen diameter) that allows pulsation indicates an artery (whereas a vein collapses without pulsation). Light compression of the artery (e.g., at the fingertip) can enhance the perceived pulse amplitude, a phenomenon supported by our findings. Beyond confirming arterial identity, the ability to deliberately modulate and detect arterial pulsations via ultrasound probe compression could have broader clinical applications. For instance, this method might be adapted for noninvasive blood pressure estimation by noting the probe pressure at which arterial pulsations become maximal or at which they disappear: analogous to identifying mean and systolic pressures, respectively, in oscillometric cuff measurements. An ultrasound-based approach could be useful in situations where a traditional cuff is difficult to apply or yields unreliable results (such as in patients with obesity, during certain surgical procedures, or at central arterial sites like the carotid artery). Moreover, the same probe compression technique can be applied to different arterial locations (e.g., radial, femoral, or carotid arteries) to assess whether similar pulsation amplitude–pressure patterns occur. Consistency of this phenomenon across multiple sites would support the broader applicability of our findings and could potentially aid in evaluating arterial stiffness or diagnosing peripheral arterial disease.

As a perspective for future research, integrating probe compression-derived arterial pulsation metrics with echocardiographic indices may enable a more comprehensive assessment of systemic hemodynamics, particularly in elderly individuals, for whom conventional evaluations are often limited.^[13]^

### 4.4. Study limitations

This study involved only healthy adult volunteers; therefore, the findings may not be generalizable to other populations, such as older adults, children, or individuals with atherosclerosis. Further studies are needed to confirm whether similar results can be observed in these groups. However, since the oscillometric blood pressure principle is also applied in these populations, albeit with alterations in the arterial pressure–area relationship, the underlying mechanism described here is likely broadly applicable.

## 5. Conclusion

Slow compression of an artery by an ultrasound probe induces pulsatile oscillations in the probe pressure that match the arterial pulsation observed on the ultrasound. The timing of the maximum amplitude pulses was the same in both measurements, mirroring the oscillometric maximum amplitude algorithm used for mean arterial pressure. These results suggest that the principles governing oscillometric blood pressure measurement and ultrasound-observed arterial pulsation are common: decreasing arterial transmural pressure by external compression and the accompanying pulsatile flow, following the pressure–volume relationship of the artery, produces the observed pulsation dynamics. To our knowledge, this is the first study to directly visualize and confirm oscillometric-like arterial pulsation behavior under localized ultrasound probe compression. These novel findings bridge ultrasound imaging and oscillometric blood pressure principles, providing a unique perspective for noninvasive arterial hemodynamic assessment.

## Acknowledgments

We would like to thank Editage (www.editage.jp) for English language editing.

Parts of the English translation of this manuscript were prepared with the assistance of an artificial intelligence tool (ChatGPT, OpenAI). The authors reviewed and edited the content to ensure accuracy and bear full responsibility for the final version of the text.

## Author contributions

**Conceptualization:** Takuya Ito, Ryohei Matsui, Hiroshi Sasano.

**Formal analysis:** Takuya Ito, Hiroshi Sasano.

**Investigation:** Marechika Tsubouchi.

**Writing – original draft:** Takuya Ito.

**Writing – review & editing:** Mami Tsubota, Yota Yamagishi, Tomonori Hattori.
